# Identification of Stress States in Compressed Masonry Walls Using a Non-Destructive Technique (NDT)

**DOI:** 10.3390/ma13122852

**Published:** 2020-06-25

**Authors:** Radosław Jasiński

**Affiliations:** Department of Building Structures and Laboratory of Civil Engineering Faculty, Silesian University of Technology, ul. Akademicka 5, 44-100 Gliwice, Poland; radoslaw.jasinski@polsl.pl; Tel.: +48-32-237-1127

**Keywords:** masonry structures, autoclaved aerated concrete masonry units (AAC), compressive strength, minor-destructive (MDT) techniques, non-destructive techniques (NDT), ultrasonic testing, acoustoelastic effect (AE)

## Abstract

The structure safety can be assessed, but only indirectly, by identifying material properties, geometry of structures, and values of loads. The complete and comprehensive assessment can be done only after determining internal forces acting inside structures. Ultrasonic extensometry using an acoustoelastic effect (AE) is among the most common non-destructive techniques (NDT) of determining true stresses in structures. Theoretical bases of the method were described in the mid 20th century. They were founded on the correlation between ultrasonic waves and the value and direction of stresses. This method is commonly used to determine stresses mainly in homogeneous materials without any inherent internal defects. This method is rarely applied to porous or composite materials, such as concrete or rock due to a high dispersion of results. Autoclaved aerated concrete (AAC), characterized by high homogeneity and porosity, is the popular material in the construction sector, used to produce masonry units. The discussed tests involved the acoustoelastic effect to determine stresses in the masonry wall made of AAC. This paper presents a widely theoretical background for the AE method, and then describes the author’s own research on AAC divided into two stages. At first, the empirical relationships between compressive stress and velocity of longitudinal ultrasonic wave, including humidity, were determined. In stage II, nine masonry walls were tested in axial compression. Mean compressive stresses in the masonry wall determined with the proposed method were found to produce a satisfactory confidence level up to ca. 50% of failure stresses. Results were significantly understated for stresses of the order of 75% of failure stresses.

## 1. Introduction

The ultrasonic technique is used for many purposes, but the most common purpose is diagnostic [1,2,3,4]. Ultrasounds are employed by many branches of the industry; they are a crucial tool for electronic engineering, telecommunications or material engineering. Generally, the application of ultrasounds is broad and covers active and passive uses. The passive use includes ultrasonic spectroscopy and defectoscopy, ultrasonic diagnostic for medical purposes, and hydrolocation. Ultrasonic waves are more and more often used to test kinetics of hardening of different types of substances. The active use includes ultrasonic coagulation and dispergation, ultrasound therapy, cavitation, development of sonoluminescence, or chemical reactions. Other active applications are: crushing and forming hard media, bonding, soldering, washing, extracting, and drying of substances. They are also used quite commonly to measure stresses in metal constructions. These methods have been elaborated to measure stresses caused by thermal treatment of rolled profiles or during welding. Ultrasonic stress measurements are based on the acoustoelastic effect (AE), that is, on the dependence of acoustic wave velocity on stress. Measurements of stress in bolts are the oldest application of the AE method. Stress is determined on the basis of measured change in the times of flight of ultrasonic waves propagating along the stressed bolt. New tests and applications related to measurements of stresses in rails, train wheels and unit shafts, have been presented in [4].

Non-destructive techniques are used for other popular materials, such as concrete or ceramics, to determine time of set, and changes in the modulus of elasticity. Ultrasonic testing using minor-destructive techniques (MDT) can determine compressive strength [5,6]. Classic methods of damage detection have been intensively developed [7,8,9,10]. No attempts have been so far made to determine stress state in materials with porous structure, such as concrete or rock. These materials behave advantageously under compression, and the complex structure of ordinary concrete cause difficulties in interpreting the results. This aspect is completely different for autoclaved aerated concrete (AAC), whose compressive strength is relatively low and at the same time this material is more homogeneous than concrete despite its porous structure.

Autoclaved aerated concrete (AAC) contains cement, calcium, and lime as binding material, sand used as a filler and tiny quantities of aluminium powder (or paste) which is used as a blowing agent. Density of this type of concrete ranges from 300 to 1000 kg/m^3^, and its compressive strength varies from 1.5 to 10 N/mm^2^. AAC has been commonly used since the middle of the 1950s. This material (>40% of the construction segment in Europe) is used for masonry structures, precast wall or floor elements, and lintels [11]. The open-pore structure explains why AAC is sensitive to direct exposure to moisture, which results in worse insulating and strength properties. The available articles, apart from general relationships specified in standards, do not contain detailed references expressed as empirical relations to determine strength properties of AAC using NDT and semi-NDT techniques. This work describes the practical application of selected issues on ultrasonic testing presented in the papers [12,13].

This paper is an attempt to evaluate changes in stress state of masonry units made of autoclaved aerated concrete built into small fragments of the masonry wall under axial compression. The masonry wall was also made of autoclaved aerated concrete with considerably more porous structure than ordinary concrete. The aim of the tests was to define empirical relationships concerning values of vertical stresses in the AAC masonry wall including the acoustoelastic (AE) effect well-known in practice [14]. This paper is divided into the theoretical part containing detailed bases of the AE method, and the research part consisting of stages I and II In stage I of the tests, experiments were performed on 24 small cube specimens (100 × 100 × 100 mm) of autoclaved aerated concrete with nominal densities of 400, 500, 600, and 700 kg/m^3^. The elastooptic constant *β*_111_ was determined that showed the longitudinal wave *c*_p0_ depended on stress *σ*_33_. In stage II, nine small models made of autoclaved aerated concrete with nominal density of 500 kg/m^3^ were prepared and tested in the second phase of tests. They were used to measure velocity of the ultrasonic wave c_p_. Relationships determined in the first phase were used to identify the stress state in the masonry wall and validate *σ*_33_—*c*_p_ relationship.

## 2. Theoretical Basis

### 2.1. Propagation of Ultrasonic Waves in Linear-Elastic Material

Generally, an anisotropic body, e.g., crystal of defined symmetry, can be the solid medium. The propagation of waves in the anisotropic medium, particularly velocity, depends on the direction relative to the axis of coordinates usually related to the crystallographic arrangement that corresponds to the given symmetry. Hooke’s law [15] describes elastic properties of the anisotropic arrangement in the linear relationship between the stress tensor *σ*_ij_ and the deformation tensor ε_kl_ in the following way:

(1) $$\sigma_{ij}=c_{ijkl}\varepsilon_{kl}+c_{ijklmn}\varepsilon_{kl}\varepsilon_{mn}+\ldots$$

where: *σ*_i_—components of stress state, *ε*_kl_—components of deformation state.

Both quantities are symmetric tensors of second rank, which means they can have six independent components. Coefficients *c*_ijkl_ and *c*_ijklmn_ are constants of elasticity of second or third rank, respectively. They are symmetric tensors of fourth and sixth rank, respectively. The linear theory of elasticity assumes materials are elastic, and the relationship between stress and deformation is linear. All constants of elasticity of third order or higher are neglected. Even for such a simplification, the number of tensor components *c*_ijkl_ defining elastic properties is 36, but the number of independent components is 21. In the case of orthotrophic materials with three mutually perpendicular planes of symmetry, elastic properties are described by nine independent constants of elasticity in the following form:

(2) $$c_{ij}=\left[\begin{array}{cccccc} c_{11} & c_{12} & c_{13} & 0 & 0 & 0\\ c_{12} & c_{22} & c_{23} & 0 & 0 & 0\\ c_{13} & c_{23} & c_{33} & 0 & 0 & 0\\ 0 & 0 & 0 & c_{44} & 0 & 0\\ 0 & 0 & 0 & 0 & c_{55} & 0\\ 0 & 0 & 0 & 0 & 0 & c_{66} \end{array}\right]$$

Regarding isotropic materials with the infinite number of axes of symmetry planes, elastic properties can be comprehensively described by two independent constants of elasticity *c*_12_ and *c*_44_. Other matrix coefficients (2) can be expressed as linear combinations using the Lamé coefficients:

(3) $$c_{11}=c_{22}=c_{33}=\lambda +2\mu ,\;c_{12}=c_{23}=c_{23}=\lambda ,\;c_{44}=c_{55}=c_{66}=\mu .$$

The force acting on any volume element in the solid medium, in which the disturbance is observed, can be expressed as the gradient of stress caused by the disturbance [1]. The Equation of the particle motion representing the equilibrium state between the restoring force and the inertial force is expressed by the following Equation:

(4) $$\rho_{0}\frac{\partial^{2}\xi_{i}}{\partial t^{2}}=\frac{\partial T_{ij}}{\partial x_{j}}\to \rho_{0}\frac{\partial^{2}\xi_{i}}{\partial t^{2}}=c_{ijkl}\frac{\partial^{2}\xi_{k}}{\partial x_{j}\partial x_{l}}$$

where: *ρ*_0_—density of the body in the tensionless state. The expression (4) contains the equations of three components of the displacement, which describe components of the wave equation of vector quantity *ξ* described by three components. Assuming that coordinates of the plane harmonic wave are expressed by the relationship $\xi =\xi_{0}e^{i\left(\omega t-kr\right)}$, Equation (4) can be expressed as:

(5) $$-\omega^{2}\rho_{0}\xi_{0i}=-c_{ijkl}k_{j}k_{l}\xi_{0k}\to \left(c_{ijkl}k_{j}k_{l}-\delta_{ik}\omega^{2}\rho_{0}\right)\xi_{ok}=0$$

where: *ω* is the wave frequency, *k_j_*, *k_l_*,—wave vector (towards *j, l*), *ξ*_0i_, *ξ*_0k_—coordinates of the plane harmonic wave (towards *i*, *k*).

Expression (5) is the system of homogeneous algebraic equations, which due to unknown *ξ*_0k_ is described in the following form:

(6) $$\begin{array}{l} \left(c_{1j1l}k_{i}k_{l}-\omega^{2}\rho_{0}\right)\xi_{10}+c_{1j2l}k_{i}k_{l}\xi_{20}+c_{1j3l}k_{i}k_{l}\xi_{30}=0\\ c_{2j1l}k_{i}k_{l}\xi_{10}+\left(c_{2j2l}k_{i}k_{l}-\omega^{2}\rho_{0}\right)\xi_{20}+c_{2j3l}k_{i}k_{l}\xi_{30}=0\\ c_{3j1l}k_{i}k_{l}\xi_{10}+c_{3j2l}k_{i}k_{l}\xi_{20}+\left(c_{3j21}k_{i}k_{l}-\omega^{2}\rho_{0}\right)\xi_{30}=0 \end{array}$$

The system of equations is fulfilled when the determinant of the coefficients is equal to 0. The equation of third degree relevant to *ω*^2^ is the solution for the determinant. The equation contains three roots that correspond to three different waves with mutually perpendicular displacements. When the simplest case of the isotropic body and waves travelling along one axis (x_3_), the determinant of the Equation (6) takes the following form:

(7) $$\left|\begin{array}{ccc} c_{44}k^{2}-\omega^{2}\rho_{0} & 0 & 0\\ 0 & c_{44}k^{2}-\omega^{2}\rho_{0} & 0\\ 0 & 0 & c_{11}k^{2}-\omega^{2}\rho_{0} \end{array}\right|=0$$

By solving the determinant, the following equation is obtained:

(8) $${\left(c_{44}k^{2}-\omega^{2}\rho_{0}\right)}^{2}\left(c_{11}k^{2}-\omega^{2}\rho_{0}\right)=0$$

It has two roots equal to $\omega_{1}^{2}=\omega_{2}^{2}=\frac{c_{44}k^{2}}{\rho_{0}}$, and the third one equal to $\omega_{3}^{2}=\frac{c_{11}k^{2}}{\rho_{0}}$. Taking into account that k=ω/C (where *C* is wave velocity), the following roots are obtained:

(9) $$C_{1}=C_{2}=\sqrt{\frac{c_{44}}{\rho_{0}}},\;C_{3}=\sqrt{\frac{c_{11}}{\rho_{0}}}$$

A solution to this issue indicates the propagation of three waves in the body. Two of them are characterized by mutually perpendicular oscillations and the same velocity *C*_1_ = *C*_2_ = *c_T_* is known as transverse waves as *c*_44_ is the shear. The third wave with the velocity *c*_p_ is the longitudinal wave because *c*_11_ is constant related to the component of the normal deformation. Taking into account relationships between material constants, the following expression is obtained:

(10) $$c_{T}=\sqrt{\frac{\mu }{\rho_{0}}},\;c_{p}=\sqrt{\frac{\lambda +2\mu }{\rho_{0}}}$$

Constants *λ* and *μ* can be introduced into the system of Equations (4) by replacing coefficients *c*_ijkl_. Then, the system of equations broken down into components is for the isotropic body as follows:

(11) $$\begin{array}{l} \rho_{0}\frac{\partial^{2}\xi_{1}}{\partial t^{2}}=\left(\lambda +2\mu \right)\frac{\partial^{2}\xi_{i}}{\partial x_{1}\partial x_{i}}+\mu \frac{\partial^{2}\xi_{1}}{\partial x_{1}\partial x_{i}}\\ \rho_{0}\frac{\partial^{2}\xi_{2}}{\partial t^{2}}=\left(\lambda +2\mu \right)\frac{\partial^{2}\xi_{i}}{\partial x_{2}\partial x_{i}}+\mu \frac{\partial^{2}\xi_{2}}{\partial x_{2}\partial x_{i}}\\ \rho_{0}\frac{\partial^{2}\xi_{3}}{\partial t^{2}}=\left(\lambda +2\mu \right)\frac{\partial^{2}\xi_{i}}{\partial x_{3}\partial x_{i}}+\mu \frac{\partial^{2}\xi_{2}}{\partial x_{2}\partial x_{i}} \end{array}$$

When the medium is incompressible (no changes in volume), the above equations give the wave equation for transverse waves in the following vector form:

(12) $$\frac{\partial^{2}\xi }{\partial t^{2}}=\frac{\mu }{\rho_{0}}\nabla^{2}\xi$$

where $\nabla^{2}$ is the Laplace operator of the second order in *n-* dimensional Cartesian coordinate system expressed as: $\nabla^{2}=\Delta =\frac{\partial^{2}}{\partial x_{i}^{2}}+\frac{\partial^{2}}{\partial x_{j}^{2}}+\frac{\partial^{2}}{\partial x_{k}^{2}}+\ldots +\frac{\partial^{2}}{\partial x_{n}^{2}}$.

Assuming the irrotational medium, the wave equation for longitudinal waves is as follows:

(13) $$\frac{\partial^{2}\xi }{\partial t^{2}}=\frac{\lambda +2\mu }{\rho_{0}}\nabla^{2}\xi$$

### 2.2. Propagation of Ultrasonic Waves in Porous Material

Biot is regarded as the initiator of works on the theory and studies on ultrasonic waves in porous materials [16,17]. According to the theory, there are two compressional waves in the wet porous material—P1-wave (the fast wave) and P2-wave (the slow wave). Further works [18,19] have confirmed Biot’s hypothesis. Other research works refer to other phenomena, including reflections and refractions, which are significant for testing and diagnosing materials. Currently, different aspects concerning wave propagation in the porous medium are examined. The issue of wave propagation and scattering in the inhomogeneous material is presented in, inter alia, the papers [20,21]. The works [22,23] present the mathematical model of propagation of low-frequency surface waves–the Stoney waves, in the porous material. Another paper [24] describes experiments on absorption and propagation of ultrasonic waves in materials with dual porosity, whereas the work [25] demonstrates test on the propagation of Rayleigh waves at liquid–solid interfaces.

Concrete, like rock media, is not ideally elastic. Therefore, the wave equation cannot be directly applied to this medium (12). The imperfect elasticity of concrete causes internal friction that transforms a part of energy into heat causing scattering and dispersion of velocity of elastic waves. The mathematical presentation of imperfection of the elastic medium is described in different ways. For example, the equation of the perfectly elastic medium can be replaced with the system of equations describing stresses and deformations. The equation of the plane longitudinal wave moving and scattered in the imperfectly elastic medium takes the following form:

(14) $$\rho_{0}\frac{\partial^{2}\xi }{\partial t^{2}}=\frac{1}{\beta_{ad}}\frac{\partial^{2}\xi }{\partial x^{2}}+\eta \frac{\partial^{3}\xi }{\partial x^{2}\partial t}$$

where *β*_ad_—adiabatic compressibility coefficient, $\eta =\eta^{\prime \prime }+\frac{4}{3}\eta^{\prime }$—viscosity coefficient composed of coefficients (*η*″) of bulk and shear viscosity (*η*′).

Generally, the solution to the wave Equation (14) is expressed as:

(15) $$\xi \left(x,t\right)=Ae^{-\alpha_{\eta }x}e^{i\omega \left(t-\frac{x}{c}\right)}+Be^{\alpha_{\eta }x}e^{i\omega \left(t+\frac{x}{c}\right)}$$

where: *A*, *B*—integration constants, *α*_η_—integration constant depending on the value of viscosity coefficient, *c*—wave velocity, x—coordinate of wavefront, *ω*—wave frequency.

The velocity of longitudinal waves in viscoelastic medium can be described as:

(16) $$c_{p}=\sqrt{\frac{K}{\rho_{0}}}\sqrt{\frac{2\left(1+\omega^{2}r^{2}\right)\left(\sqrt{1+\omega^{2}r^{2}}-1\right)}{\omega^{2}r^{2}}}$$

where $r=\eta \beta_{ad}$, $K=1/\beta_{ad}$.

The velocity of waves in the inhomogenuous granular medium, despite being the material constant, is related to its physical properties—density, elasticity defined by the Lamé coefficients also depends on wave scattered by the medium, wave frequencies, the medium structure, etc. Therefore, velocity not regarded as the constant value in contrast to the propagation of waves in perfectly elastic media. Granular media, such as rocks, concrete, or mortar, are characterized by:

- different dimensions and properties of components—matrix grains,
- different models (systems) of arrangement and connections of individual grains—they can have a direct contact or are connected with binder of other properties. In the case of chemically bonded materials, the binder changes its properties during the transformation from liquid to solid state.

Scattering of the elastic wave in granular media depends on many factors—mechanical and thermal processes caused by the propagating wave. There are three main reasons for energy loss during wave scattering:

- internal frictions in the medium—*δ*_r_,
- thermal effects—*δ*_T_,
- Rayleigh scattering *δ*_R_.

The overall wave scattering is the sum of mentioned elements:

(17) $$\delta =\delta_{r}+\delta_{T}+\delta_{R},$$

The role of each of the three factors above in ultrasonic wave scattering in the homogeneous granular medium depends on the frequency and structure of that medium characterized by:

- dimensions of the matrix grains,
- thermal properties of components,
- elastic properties of components, and their density.

Wave velocity in granular media characterized by a large coefficient of wave scattering can be calculated from the following dependence:

(18) $$C=C_{0}\sqrt{1-{\left(\frac{\delta }{\omega }\right)}^{2}}$$

where *C*_0_—wave velocity in the linear-elastic medium, *δ*—total scattering coefficient, and *ω*—wave frequency.

The velocity of wave propagation in granular materials changes within a wide range and is subjected to fluctuations depending on the type of components and their distribution. It is caused by different values of elasticity constants *E G ν* demonstrated by individual components of granular materials. Therefore, we obtain a certain mean velocity that results from the percentage contribution of velocity to individual components. Determining ultrasound velocity for different specimens cannot be neglected in that case. Greater scattering and more problems related to signal recording are expected in specimens with longer wave paths. Hence, the use in NDT methods requires the conversion of wave velocities.

## 3. Stress Measurements Using an Ultrasonic Technique

Material stress can affect velocity of the acoustic wave due to inhomogeneity and anisotropy of the material. That effect has been described for the first time by seismologist Biot [26] and experimentally verified by Hughes and Kelly [27] and Bergman and Shahbender [28]. It is demonstrated that the static stress can change velocity of the acoustic wave in the medium, and that effect is called the acoustoelastic (AE) effect [29,30].

The acoustoelastic effect is based on the relationship between the velocity of transverse wave propagation and stress in solid bodies found by Benson and Raelson in the 1970s [14]. Since then, this aspect has been widely developed [31,32,33]. The impact of stress on the velocity of transverse wave propagation is determined by the direction of wave propagation with reference to the stress direction and wave polarization. A change in the polarization plane depends on stress, similarly to a light wave in the elastooptic effect. Its mechanism was theoretically described on the basis of the non-linear theory of solid deformation [27]. According to that theory, constant elasticity of higher orders (than those observed in the theory of linear elasticity) was responsible for nonlinear effects. The propagation velocity in the stressed body can be expressed as the sum of velocities in the tensionless stress (*σ* = 0) and its change (increment) caused by stress. That change can be defined as dependent on stress including constant characteristics of elasticity of second or third order.

In accordance with the infinite deformation of elastic materials by Murnaghan [34], the stress-deformation relationship should be described by the function of free energy *W*_s_ defined as [27,35]:

(19) $$W_{s}=\frac{1}{2}\left(\lambda +2\mu \right)I_{1}^{2}-2\mu I_{2}+\frac{1}{3}\left(l+2m\right)I_{1}^{3}-2mI_{1}I_{2}+nI_{3}$$

where: *λ, μ*—Lamé constants, *l m n*—elasticity constants of second and third order by Murnaghan, *I*_1_, *I*_2_, *I*_3_—deformation invariants.

Taking into account the principle of energy conservation, Hooke’s law can be expressed as:

(20) $$\rho \delta W_{s}=\sigma_{ij}\frac{\partial \delta u_{i}}{\partial u_{j}},$$

where *δW* and *δu_i_* mean finite increments in the function of free energy and displacement area, *ρ* is density after deformation. The combination of Equations (19) and (20) produces the acoustoelastic equation, which binds the static load with velocity of the elastic wave under hydrostatic pressure *P*:

(21) $$\begin{array}{l} \rho_{0}c_{p}^{2}=\lambda +2\mu -\frac{P}{3\lambda +2\mu }\left(6l+4m+7\lambda +10\mu \right)\;,\\ \rho_{0}c_{T}^{2}=\mu -\frac{P}{3\lambda +2\mu }\left(3m+0,5n+3\lambda +6\mu \right)\;, \end{array}$$

where: *c*_p_ and *c*_T_ are velocity of longitudinal and transverse waves respectively, a *ρ*_0_—body density in the tensionless state.

**Figure 1:**
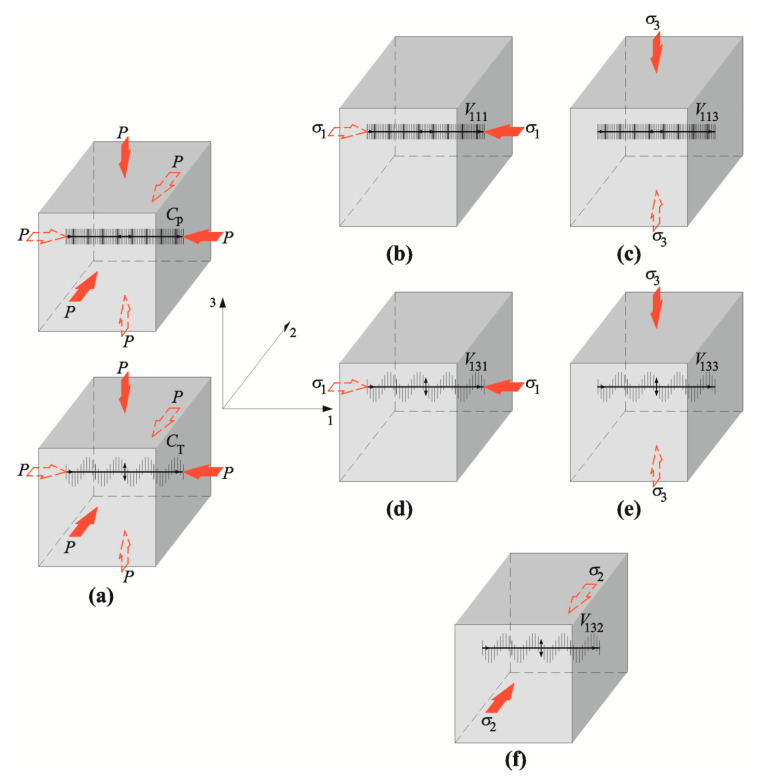
Identification of velocity of ultrasonic waves in isotropic material: (**a**) hydrostatic compression by pressure P, (**b**) longitudinal wave under stress *σ*_1_, (**c**) transverse wave under stress *σ*_3_, polarized in planes 1–3 (**d**) transverse plane under stress *σ*_1_, polarized in planes 1–3, (**e**) transverse plane under stress *σ*_3,_ polarized in planes 1–3, (**f**) longitudinal wave under stress *σ*_2,_ polarized in planes 1–3.

Thus, the hydrostatic level of stress can be defined from Equation (20) [36] by measuring velocity of the longitudinal and transverse waves—Figure 1a. In the case of uniaxial stress, wave velocity depends on the direction of the stress and the square of velocity on Figure 1b-1f is as follows:

(22) $$V_{111}^{2}=\frac{\lambda +2\mu }{\rho_{0}}-\frac{\sigma_{1}}{3K_{0}\rho_{0}}\left[\frac{\lambda +\mu }{\mu }\left(4\lambda +10\mu +4m\right)+\lambda +2l\right],$$

(23) $$V_{113}^{2}=\frac{\lambda +2\mu }{\rho_{0}}+\frac{\sigma_{3}}{3K_{0}\rho_{0}}\left[\frac{2\lambda }{\mu }\left(\lambda +20\mu +m\right)-2l\right],$$

(24) $$V_{131}^{2}=\mu -\frac{\sigma_{1}}{3K_{0}\rho_{0}}\left[4\lambda +4\mu +m+\frac{\lambda n}{4\mu }\right],$$

(25) $$V_{133}^{2}=\mu -\frac{\sigma_{3}}{3K_{0}\rho_{0}}\left[\lambda +2\mu +m+\frac{\lambda n}{4\mu }\right],$$

(26) $$V_{132}^{2}=\mu +\frac{\sigma_{2}}{3K_{0}\rho_{0}}\left[2\lambda -m+\frac{n}{2}\frac{\lambda }{2}\frac{n}{\mu }\right],$$

where: $K_{0}=\frac{E}{3\left(1-2\nu \right)}=\frac{2\mu +3\lambda }{3}$.

Knowing velocity of the ultrasonic wave in the loaded material and elasticity constants of the first (*λ μ*), second and third order (*m n l*) normal stresses can be determined. Measurements of wave velocity do not cause any problems except for small specimens (due to high sensitivity of the recording equipment). However, determining material constants *m, n,* and *l* is difficult.

Using the equation [27], the precise method of determining material constants was presented in the papers [37,38]. Velocities of longitudinal and transverse waves under the uniaxial stress are presented in the following form:

(27) $$\rho_{0}V_{11}^{2}=\lambda +2\mu +\frac{\sigma_{1}}{E}\left[5\lambda +10\mu +2l+4m-2\nu \left(\lambda +2l\right)\right]\to V_{11}^{2}=V_{0}^{2}\left(1+2\alpha_{11}\frac{\sigma_{1}}{E}\right)$$

(28) $$\rho_{0}V_{12}^{2}=\mu +\frac{\sigma_{1}}{E}\left[\lambda +4\mu +m-\nu \left(2\lambda +2\mu +2m-\frac{n}{2}\right)\right]\to V_{11}^{2}={\left(\frac{\mu }{\rho_{0}}\right)}^{2}\left(1+2\alpha_{12}\frac{\sigma_{1}}{E}\right)$$

(29) $$\rho_{0}V_{21}^{2}=\mu +\frac{\sigma_{1}}{E}\left[\lambda +\mu +m-\nu \left(2\lambda +4\mu +2m-\frac{n}{2}\right)\right]\to V_{21}^{2}={\left(\frac{\mu }{\rho_{0}}\right)}^{2}\left(1+2\alpha_{21}\frac{\sigma_{1}}{E}\right)$$

(30) $$\rho_{0}V_{22}^{2}=\lambda +2\mu +\frac{\sigma_{1}}{E}\left[\lambda +2l-\nu \left(6\lambda +10\mu +4l+4m\right)\right]\to V_{22}^{2}=V_{0}^{2}\left(1+2\alpha_{22}\frac{\sigma_{1}}{E}\right)$$

(31) $$\rho_{0}V_{23}^{2}=\mu +\frac{\sigma_{1}}{E}\left[\lambda +m-\frac{n}{2}-\nu \left(2\lambda +6\mu +2m\right)\right]\to V_{21}^{2}={\left(\frac{\mu }{\rho_{0}}\right)}^{2}\left(1+2\alpha_{23}\frac{\sigma_{1}}{E}\right)$$

Tests on the specimens of two lengths were the base to formulate relationships for determining constants *α*_11_, *α*_22_, *α*_12_, *α*_21_, and *α*_23_ from the following equations:

(32) $$\alpha_{11}=1-\frac{E}{\sigma_{1}}\left[\frac{L_{1}}{L_{2}-L_{1}}\left(\frac{\Delta t_{1}}{t_{01}}\right)-\frac{L_{1}}{L_{2}-L_{1}}\left(\frac{\Delta t_{2}}{t_{02}}\right)\right]$$

where: *L*_1_—length of specimens ”1” and “2” used for calibration, Δ*t*_1_ = *t*_1_ − *t*_01_—difference in passing time of wave in specimen “1” after deformation (*t*_01_) and before deformation (*t*_01_), Δ*t*_2_ = *t*_2_ − *t*_02_—difference in passing time of wave in specimen “2” after deformation (t_02_) and before deformation (*t*_02_).

The equation for determining other material constants is as follows:

(33) $$\alpha_{1j}=-\nu -\frac{E}{\sigma_{1}}\left(\frac{\Delta t}{t_{0}}\right)$$

where Δ*t* = *t*_1_ − *t*_01_ is the difference in passing time of the wave in the specimen after and before its deformation.

My own research indicated the linear nature of changes in the ratio of passing time of the wave Δ*t*/*t*_0_ in relation to stress increase. Determining other constants consisted in solving the following system of equations:

(34) $$l=\frac{\left(2\alpha_{11}-5\right)\left(\lambda +2\mu \right)}{2\left(1-2\nu \right)}-\frac{2m-\nu \lambda }{1-2\nu },\;m=\left[\frac{\alpha_{11}-\alpha_{22}}{2\left(1+\nu \right)}-1\right]\left(\lambda +2\mu \right)-\frac{\mu }{2}\;n_{12}=\frac{2}{\nu }\left[-\left(a+4\nu \right)+2\nu \left(a+\mu \right)+2\mu \alpha_{12}\right],\;n_{21}=\frac{2}{\nu }\left[-\left(a+2\nu \right)+2\nu \left(a+2\mu \right)+2\mu \alpha_{21}\right]\;n_{23}=2\left[a-2\nu \left(a+3\mu \right)-2\mu \alpha_{23}\right]$$

where: *a* = *λ* + *m*.

**Figure 2:**
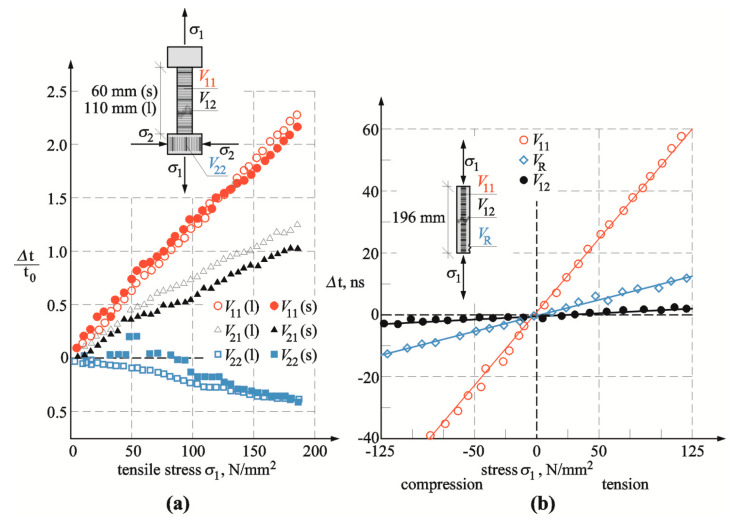
Selected test results for acoustoelastic effect: (**a**) ratio of changes in velocity of waves of different length obtained from tests (adapted from [38]), (**b**) changes in velocity of longitudinal, transverse, and Rayleigh waves obtained from tests (adapted from [40]).

Figure 2 shows changes in increment of propagation time of longitudinal and transverse waves described in the paper by Takahashi [38], who based this on his experience of developing and patenting the measuring apparatus to determine directly constants *l, m,* and *n* [39].

As expected, the greatest increments in wave velocity were observed for longitudinal waves in the direction of stress. In addition, surface waves could be used to detect changes in stress states. The smallest gradients of velocity were obtained for transverse waves. As expected, the greatest increments in wave velocity were observed for longitudinal waves perpendicular to the stress direction. An increase in compressive stress caused an increase in wave velocity. Similar relationships were observed for waves propagating perpendicularly to the stress direction. Theoretical principles of the acoustoelastic effect are relatively well documented in the literature. There is also an apparatus to determine elasticity constants *l, m,* and *n* of the third order for metals and plastic in accordance with procedures described in, among others, papers [37,38,41]. Diagnosing stress states in structures using the NDT method requires the information on load direction and defined gradient of changes in velocity of longitudinal or transverse wave Knowing Muraghan coefficients is not essential. 

## 4. Test Program and Results

The test program was divided into two stages. Stage I included the material tests on specimens made of autoclaved aerated concrete (AAC) to determine density ρ_0_, elasticity modulus E and Poisson’s ratio υ. Each cube specimen was subjected to axial compression until the failure and velocity of the longitudinal wave were determined at different normal stresses. The obtained results were used to determine linear correlations describing a σ–C_p_ relationship. In stage II, nine models of masonry walls were tested in axial compression. The velocity of the longitudinal wave was measured at different values of vertical loads. Then, vertical loads were determined on the basis of a correlation curve obtained during stage I. To interpret the results, they were compared with numerical calculations for 3D models of the masonry wall. 

### 4.1. Stage I—Determination of Acoustoelastic Constant

The tests included four series of masonry units with thickness within the range of 180–240 mm and different classes of density: 400 kg/m^3^, 500 kg/m^3^, 600 kg/m^3^, and 700 kg/m^3^, each 20 masonry units were randomly selected. Six cores with a diameter of 59 mm and the height of 120 mm were taken from each type of the masonry unit using a drill. They were used to determine fundamental properties of tested autoclaved aerated concrete (AAC). All drilled cores were dried until constant weight at temperature of 105 ± 5 °C. Then, two vertical and horizontal electro-resistant tensometers were fixed to side surfaces of cylindrical specimens to measure deformations and determine modulus of elasticity E within the range of 0.1–0.33 *σ*_max_ and Poisson’s ratio υ at the level of 0.33 *σ*_max_. Tests were conducted using the testing machine, in which an increment in load was controlled manually, and the reading range of the dynamometer was 100 kN. Mean mechanical parameters obtained for all tested types of masonry units are shown in Table 1. The presented results from testing density were taken from the paper [12].

Besides the cores used to determine properties of AAC, four series of six cuboid specimens each (24 specimens in total) were drilled using a diamond saw 4. The specimens had dimensions of 100 × 100 × 100 mm, and were used as basic specimens for determining the strength *f*_B_ in accordance with Appendix B to the standard EN 771-4 [42]) harmonized with the European standard PN-EN 1996-1-1:2010 [43].

All specimens drilled from blocks to determine the correlation between vertical stresses and ultrasound velocity, were air-dried until constant weight at a temperature of 105 ± 5 °C (for at least 36 h). That way, the impact of moisture content on AAC was eliminated [13,44]. Generally, it tends to reduce significantly compressive strength and change velocity of the ultrasonic wave propagation [12].

**Figure 3:**
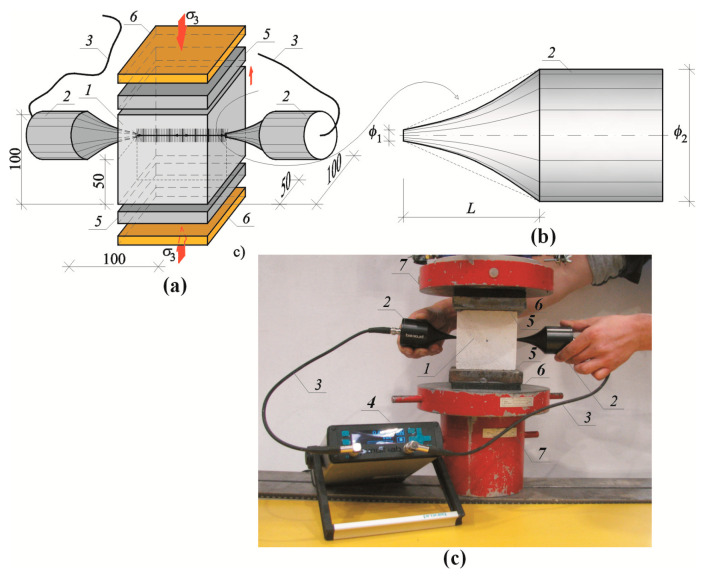
A test stand for measuring ultrasonic wave velocity in compressed specimens: (**a**) specimen geometry and elements of the stand, (**b**) geometry of exponential transducer, (**c**) a test stand; *1*—tested AAC specimen 100 × 100 × 100 mm, *2*—exponential transducers, *3*—cables connecting transducers with recording equipment, *4*—recording equipment, *5*—steel sheet, 15 mm thick, 6—vibration isolation, *7*—heads of testing machine.

The ultrasonic technique, commonly applied to test strength of concrete and masonry, was used to determine velocity of ultrasonic waves in AAC [45,46]. Ultrasonic testing was conducted on the block specimens 100 × 100 × 100 mm drilled from masonry units—Figure 3. The specimens in air-dry conditions and relative humidity w/wmax = 0% were used for testing. Each series of elements included at least six specimens, and 24 specimens in total were tested. PUNDIT LAB (Proceq SA, Schwerzenbach, Switzerland) instrument was used for tests. Exponential transducers with the waveguide length *L* = 50 mm, diameters ø1 = 4.2 mm and ø2 = 50 mm, and frequency 54 kHz were employed. The measurement accuracy of passing time of the ultrasonic wave was equal to ±0.1 μs. The used methodology of testing and equipment were typical for ultrasonic tomography for concrete and masonry [47,48]. Each specimen was placed on transducers of the testing machine (type FORM+TEST Prüffsysteme MEGA 3 with the range of 100 kN, class 1, reading accuracy ±1%) through the vibration isolation washer and steel sheet of 3 mm thickness. The steel plate and the vibration isolation washer were placed on the top surface of the specimen. Vibration isolation was necessary for eliminating possible vibrations that could affect the results from measurements of ultrasonic waves. Then, the transducers were applied to opposite walls and the passing time of wave was measured using the transmission method. The transducers were in contact with the specimens at an angle of 90° within distance between the transducers measured every time with accuracy up to 1 mm. The tests were conducted for various loading of the specimens and the force was scaled every 2.5 kN.

**Figure 4:**
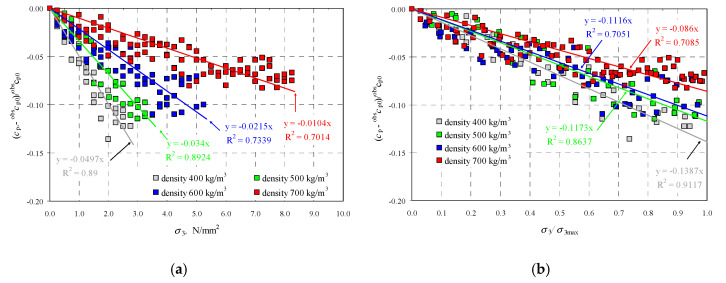
Results from measuring velocity of the longitudinal ultrasonic wave: (**a**) relative change in velocity of longitudinal wave as a function of compressive stress, (**b**) relative change in velocity of longitudinal wave as a function of relative compressive stresses.

The selected results from measurements and maximum values of stress *σ*_3max_ are presented in Table 2. There are also empirical values of the longitudinal wave ^obs^*c*_p0_ without the participation of compressive stress and ratios of normal stress *σ*_3_/*σ*_3max_, for which the measurements are presented in a tabular form. Figure 4a illustrates results from measured velocities of ultrasounds as the ratio (*c*_p_–^obs^*c*_p0_)/^obs^*c*_p0_ expressing the relative increment in ultrasound velocity as a function of stress *σ*_3_. Relative increments in velocity of ultrasonic waves are presented in Figure 4b as a function of relative compressive stress *σ*_3_/*σ*_3max_.

As in previous tests [12], the specimens dried until constant weight demonstrated an increase in ultrasound velocity with increased density of AAC under stress *σ*_3_ = 0. Velocity ^obs^*c*_p0_ increased to 1875 m/s in concrete of a nominal class of 400 kg/m^3^, and to 2225 m/s in concrete with density of 700 kg/m^3^. Increased compressive stress in all specimens caused nearly proportional drop in ultrasound velocity. Under relatively low stress when 0 ≤ *σ*_3_ ≤ 0.25*σ*_3max_, values of ultrasound velocity decreased by 2–4% when compared to ^obs^c_p0_. When normal stress increased to the level of 0.25*σ*_3max_ ≤ *σ*_3_ ≤ 0.50*σ*_3max_, the velocity of ultrasounds decreased by 5–7% when compared to the reference value of 0.25*σ*_3max_. Under greater values of relative stress 0.50*σ*_3max_ ≤ *σ*_3_ ≤ 0.75*σ*_3max_, the greatest percentage drop in propagation of ultrasonic waves by 9–11% was found in concrete with nominal densities of 400 and 500 kg/m^3^. The reduction in velocity of ultrasonic waves by 7–9% was observed in the specimens made of concrete with density of 600 and 700 kg/m^3^. No clear reduction in wave velocity in concrete with densities of 600 and 700 kg/m^3^ was observed for the stress level, at which slight noise was heard in the specimens and local crushing was apparent within the stress range of 0.75*σ*_3max_ ≤ *σ*_3_ ≤ 0.95*σ*_3max_. The relative velocity of ultrasounds decreased by 11–12% in other specimens. In conclusion, a nearly linear drop in relative velocity of longitudinal ultrasonic wave was observed regardless of AAC density. The maximum reduction in relative velocity of ultrasounds was directly proportional to AAC density and changed within the range of 7–12%. As in tests conducted on metals [38,40], linear relationships were obtained, which defined the reduction in velocity of ultrasonic wave propagation as a function of applied normal stress. Considering the relationship (23), accurate physical relationships can be determined:

(35) $$V_{113}^{2}=V_{0}^{2}+\frac{1}{3K_{0}\rho_{0}}\left[\frac{2\lambda }{\mu }\left(\lambda +20\mu +m\right)-2l\right]\sigma_{3}\to c_{p}^{2}=c_{p0}^{2}+\frac{1}{3K_{0}\rho_{0}}\left[\frac{2\lambda }{\mu }\left(\lambda +20\mu +m\right)-2l\right]\sigma_{3}\;c_{p}^{2}-{}^{obs}c_{p0}^{2}=\left(c_{p}-c_{p0}\right)\left(c_{p}+c_{p0}\right)\approx \left(c_{p}-c_{p0}\right)2c_{p0},\;\frac{\left(c_{p}-c_{p0}\right)2c_{p0}}{c_{p0}}=\frac{1}{6K_{0}\rho_{0}c_{p0}}\left[\frac{2\lambda }{\mu }\left(\lambda +20\mu +m\right)-2l\right]\sigma_{3}\to \frac{\left(c_{p}-c_{p0}\right)}{c_{p0}}=\frac{\frac{\lambda }{\mu }\left(\lambda +20\mu +m\right)-2l}{\left(\lambda +2\mu \right)\left(3\lambda +2\mu \right)}\sigma_{3}.$$

The relationship after transformation can be expressed as:

(36) $$\frac{\left(c_{p}-c_{p0}\right)}{c_{p0}}=\frac{\left(t_{p0}-t_{p}\right)}{t_{p}}=\frac{\frac{\lambda }{\mu }\left(\lambda +20\mu +m\right)-2l}{\left(\lambda +2\mu \right)\left(3\lambda +2\mu \right)}\sigma_{3}=\beta_{113}\sigma_{3},$$

where *β*_113_ is the acoustoelastic effect [40] related to the longitudinal wave perpendicular to the direction of the applied load.

If c_p0_ in the relationship (36) is replaced with the value determined in the tests, then the relationship illustrated in Figure 4a is obtained. By dividing both sides of the Equation (36) by the value of maximum stress *σ*_3max_, the following relationship is developed:

(37) $$\frac{\left(c_{p}-c_{p0}\right)}{c_{p0}}=\frac{\left(t_{p0}-t_{p}\right)}{t_{p}}=\gamma_{113}\frac{\sigma_{3}}{\sigma_{3\max}}.$$

where *γ*_113_ = *β*_113_ *σ*_3max_ can be called the relative acoustoelastic coefficient.

**Figure 5:**
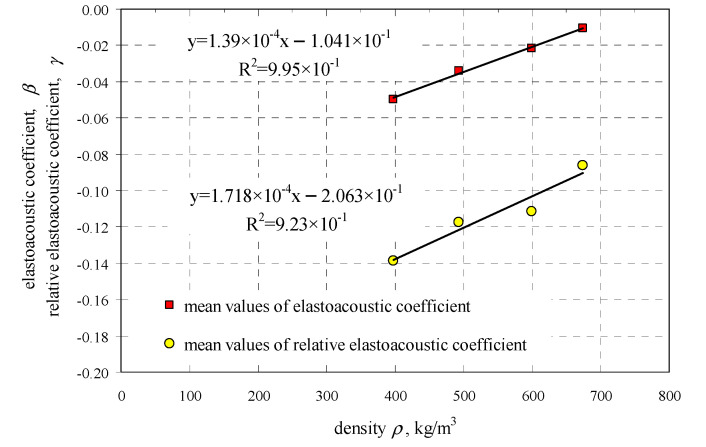
Values of coefficients β_113_ and γ_113_ as a function of AAC density.

The introduction of coefficient *β*_113_ considerably simplifies practical applications. By using relative values of passing time of the wave, the effect of wave scattering and other related effects described under point 2.2 could be neglected. If *c*_p0_ in the relationship (37) was replaced with the value determined in the tests, then the relationship illustrated in Figure 4b was obtained. It was adequate to know the coefficient *γ*_113_ to determine the maximum value of compressive stresses corresponding to normalized compressive strength of the masonry unit *f*_Bw_ in air-dry conditions. The obtained values of coefficients *β*_113_ and *γ*_113_ for straight lines determined from Equations (36) and (37) as a function of density are presented in Figure 5.

Empirical relationships developed from obtained results were proposed to express values of coefficients β_113_ and γ_113_ as a function of AAC density at (w = 0)

(38) $$\beta_{113}=1.39\times 10^{-4}\rho -0.104,\;R^{2}=0.995,\;\gamma_{113}=1.72\times 10^{-4}\rho -0.206,\;R^{2}=0.923\;\mathrm{when}\;397\;\frac{\mathrm{kg}}{m^{3}}\leq \rho \leq 674\;\frac{\mathrm{kg}}{m^{3}}$$

The practical applications required taking into account moisture content of AAC. The paper [12] demonstrated that the maximum moisture content in concrete depended on nominal density. At the density increase in the range from *ρ* = 397 kg/m^3^ to 674 kg/m^3^, the maximum moisture content was varying within *w*_max_ = 53.3–89.9%, which made it possible to determine a straight line of the least square in the following form:

(39) $$w_{\max}=-1.23\times \frac{\rho }{1000}+1.34,\;\mathrm{when}\;397\;\frac{\mathrm{kg}}{m^{3}}\leq \rho \leq 674\;\frac{\mathrm{kg}}{m^{3}}$$

**Figure 6:**
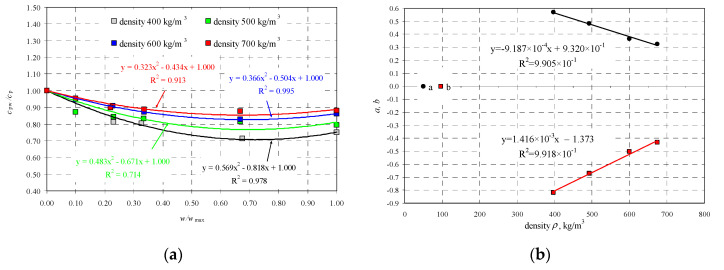
Relationship between velocity of ultrasonic wave propagation, moisture content and density: (**a**) relative changes in velocity of longitudinal wave as a function of relative moisture content w/w_max_, (**b**) values of coefficients as a function of AAC density.

Moreover, relative changes in velocity of longitudinal ultrasonic waves were shown by the relationships illustrated in Figure 6.

The tests were used to develop the following relationships including velocity *c*_pw_ in wet AAC with reference to AAC in air-dry conditions *c*_p_:

(40) $$\frac{c_{pw}}{c_{p}}=0.569\frac{w}{w_{\max}}-0.818+1,\;\mathrm{when}\;397\;\frac{\mathrm{kg}}{m^{3}}\leq \rho \leq 446\;\frac{\mathrm{kg}}{m^{3}},\;\frac{c_{pw}}{c_{p}}=0.483\frac{w}{w_{\max}}-0.671+1,\;\mathrm{when}\;462\;\frac{\mathrm{kg}}{m^{3}}\leq \rho \leq 532\;\frac{\mathrm{kg}}{m^{3}},\;\frac{c_{pw}}{c_{p}}=0.366\frac{w}{w_{\max}}-0.504+1,\;\mathrm{when}\;562\;\frac{\mathrm{kg}}{m^{3}}\leq \rho \leq 619\;\frac{\mathrm{kg}}{m^{3}},\;\frac{c_{pw}}{c_{p}}=0.323\frac{w}{w_{\max}}-0.434+1,\;\mathrm{when}\;655\;\frac{\mathrm{kg}}{m^{3}}\leq \rho \leq 725\;\frac{\mathrm{kg}}{m^{3}}.$$

After taking into account the obtained results, values of empirical coefficient defined the following linear relationships:

(41) $$a=9.187\times 10^{-4}\rho +0.932,\;\mathrm{when}\;397\;\frac{\mathrm{kg}}{m^{3}}\leq \rho \leq 674\;\frac{\mathrm{kg}}{m^{3}}.\;b=1.416\times 10^{-3}\rho -1.373,\;\mathrm{when}\;397\;\frac{\mathrm{kg}}{m^{3}}\leq \rho \leq 674\;\frac{\mathrm{kg}}{m^{3}}.$$

### 4.2. Stage II—Test Results for Small Masonry Models

**Figure 7:**
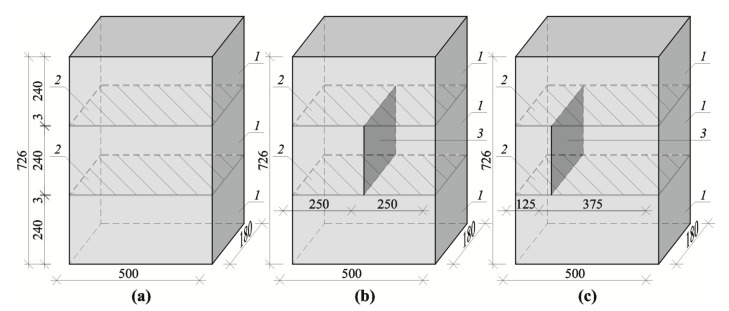
Geometry of models made of AAC tested in stage II (dimensions in mm): (**a**) models of series I without head joint, (**b**) models of series II with head joint at the mid-length of the element, (**c**) models of series III with head joint at 1/4 length; *1*—masonry units, *2*—bed joints, *3*—head joints.

Stage II consisted of verifying empirical relationships developed in stage I. Small masonry walls made of AAC of nominal type of 600 kg/m^3^, with thin joints laid in the ready-mixed mortar and with the strength *f*_m_ equal to 6.10 N/mm^2^ [49] were used for that purpose. Nine test elements in total were prepared and divided into three series marked as I, II, and III. All elements had the same external dimensions: the length of 500 mm, the height of 724 mm, and the thickness of 180 mm. The presence of the head joint or its lack differentiated the models. This was required to highlight potential effects in changes of ultrasound wave velocity in the real wall near head joints. All models of series I were made from three masonry units without the head joint. The models of series II had the head joint in the central layer at the mid-length of the masonry units, and those of series III had the head joint at 1/4 of the masonry length. The view, shape, and dimensions of tests elements of series I, II, and III are shown in Figure 7.

**Figure 8:**
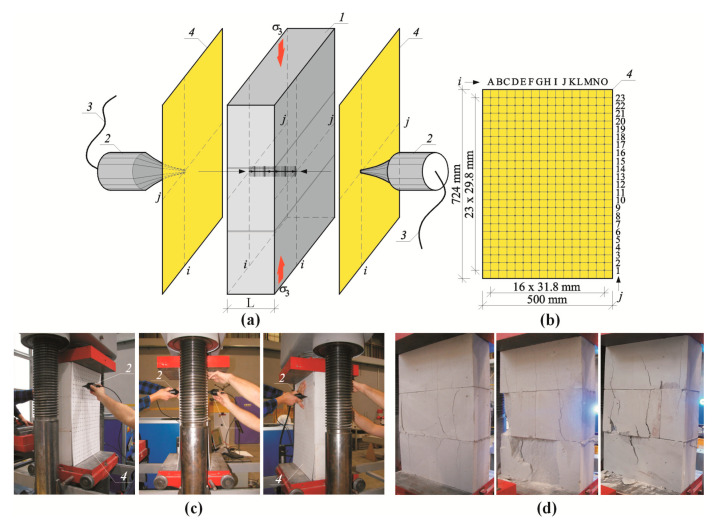
Testing methodology for models made of AAC used in stage II: (**a**) measurement of velocity of ultrasonic wave propagation at different stress values σ_3_, (**b**) template geometry used for symmetric arrangement of ultrasonic transducers, (**c**) models during tests, (**d**) failure of selected models *1*—masonry units, *2*—ultrasonic transducers, *3*—cables connecting transducers with recording equipment, *4*—templates for symmetric location of ultrasonic transducers.

**Figure 9:**
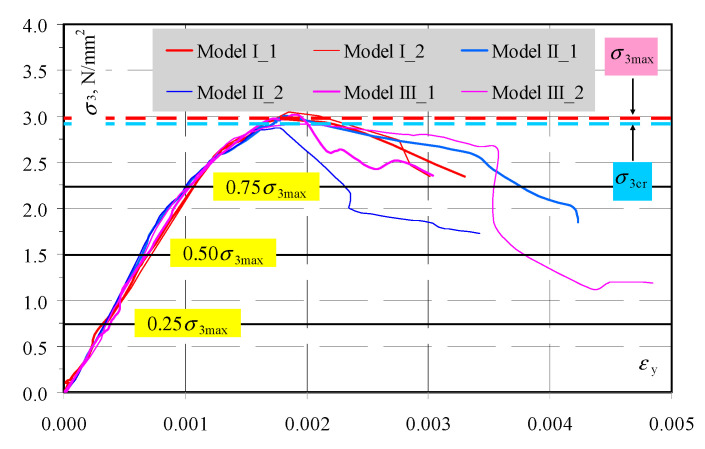
Relationships between stress and strain σ-ε for all tested models.

Test models were placed in the strength testing machine with an operating range of 1000 kN (class 1). The applied load was perpendicular to the plane of bed joints and the machine piston displacement was monotonically increasing at a rate of 1 mm/min. The value of the applied load *F* was read from the dynamometer of the testing machine. Stress applied to top and bottom parts of the bed surface of the model was calculated from the equation *σ*_3_ = *F/A* (where *A*—area of bed surface of the element *A* = 180 × 500 = 90,000 mm^2^). During the tests, displacements and deformations were measured for two models of each series with the Digital Image Correlation (DIC) using the ARAMIS 6M system by GOM GmbH Braunschweig, Germany (the class of reading accuracy for displacements was 1%) [50,51,52]. To determine values of forces and stresses causing cracks (*σ*_3cr_) and failure (*σ*_3max_), some models of each series (I-3, II-3, III-3) were tested without measuring the velocity of ultrasonic wave propagation. Wave velocity c_p_ was measured in two other models at the following values: 0, 0.25*σ*_3max_, 0.50*σ*_3max_, 0.75*σ*_3max_. The transmission method was used to measure waves. Hence, the precise arrangement of ultrasonic transducers vis-à-vis each other was necessary. For that purpose, two plastic templates were used with holes having a diameter of 5 mm, made at the regular spacing adjusted to the model geometry—Figure 8a,b. Holes in the template (Figure 8b) were placed in horizontal and vertical configuration within a distance of ~30 mm. Before testing, apparent density ρ_0_ in air-dry conditions, relative moisture content in the material used for preparing the models were calculated, and additionally the maximum moisture content w_max_ was calculated from the following relationship (39). Basic results for properties of the models and test results in the form of stresses causing cracks *σ*_3cr_, and maximum stresses *σ*_3max_ are presented in Table 3, whereas relationships between compressive stress and deformation σ-ε are illustrated in Figure 9. All models were characterized by minor differences in obtained parameters. Density of models varied from 587 to 597 kg/m^3^, and relative moisture content was within the range of 4.5–6.0%. At determined values of loading, the procedure of loading was stopped to measure passing time *t*_p_ of the ultrasonic wave, and then the propagation velocity was calculated from the relationship *c*_p_ = *L/t*_p_ (*L* = 180 mm). The tests were performed only on one model of each series (highlighted rows in Table 3). No measurements were made when the measuring points overlapped with bed or head joints.

Nearly proportional increase in deformations was observed in all models exposed to increasing loading. Clear breaking of graphs illustrating stress–deformation relationships was only observed at the time preceding maximum stress that was reached under mean stress within the range of 2.96–3.01 N/mm^2^. Cracks on external surfaces of masonry units were not observed until maximum stress that was reached in the weakening phase under mean stress within the range of 2.89–2.95 N/mm^2^.

**Figure 10:**
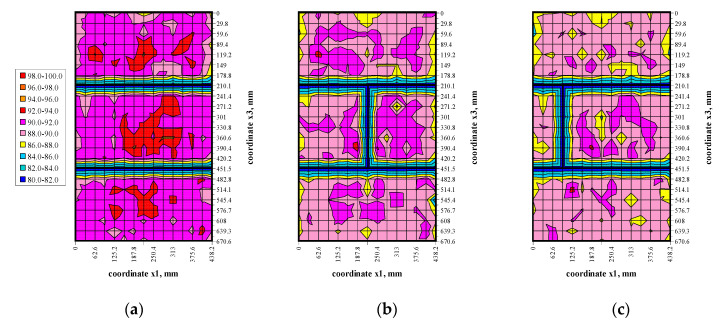
Results from measuring passing time of the ultrasonic wave under the load *σ*_3_ = 0: (**a**) model I-1, (**b**) model II-1, (**c**) model III-1.

**Figure 11:**
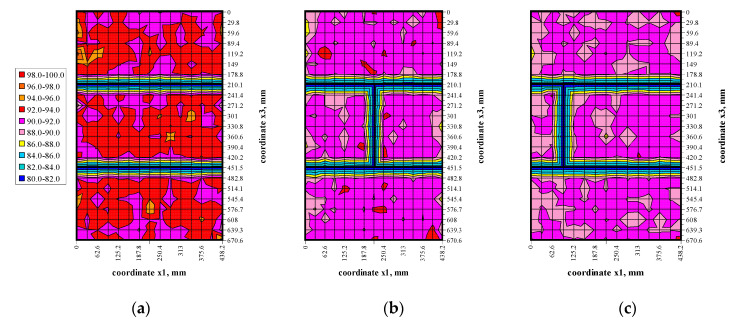
Results from measuring passing time of the ultrasonic wave under the load *σ*_3_ = 0.25*σ*_3max_: (**a**) model I-1, (**b**) model II-1, (**c**) model III-1.

**Figure 12:**
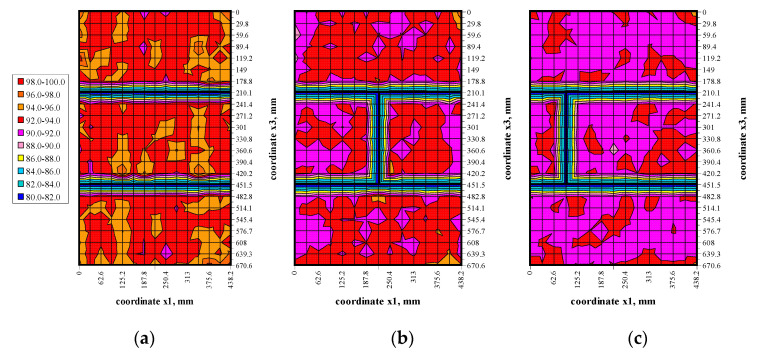
Results from measuring passing time of the ultrasonic wave under the load *σ*_3_ = 0.50*σ*_3max_: (**a**) model I-1, (**b**) model II-1, (**c**) model III-1.

**Figure 13:**
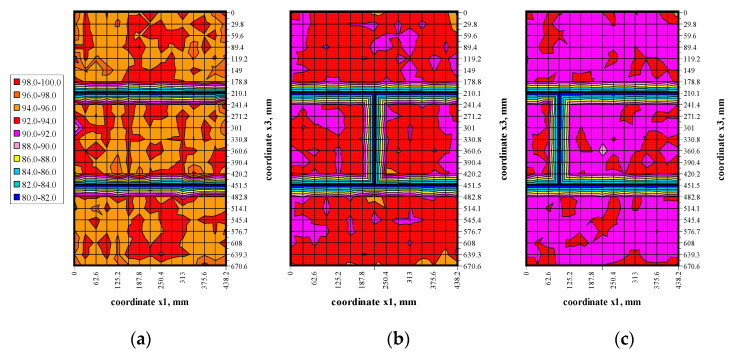
Results from measuring passing time of the ultrasonic wave under the load *σ*_3_ = 0.75*σ*_3max_: (**a**) model I-1, (**b**) model II-1, (**c**) model III-1.

The transmission method was used to measure passing time of ultrasonic wave at stress levels (0, 0.25*σ*_3max_, 0.50*σ*_3max_, 0.75*σ*_3max_) shown in Figure 9. Results in the form of maps showing passing time of the wave t_p_ are illustrated in Figure 10, Figure 11, Figure 12 and Figure 13.

Basic results in the form of mean time of wave propagation for all points are compared in Table 4.

The conducted tests indicated passing times of the ultrasonic wave in walls under zero loads were not constant, some fluctuations were observed—Figure 10. Usually, waves in central parts of the elements had the longest passing time. Clear disturbances at vertical edges and near bed joints were observed. However, the calculated coefficient of variation for all measurements, and from disturbed areas, was relatively low in the order of 1.4–1.6% due to a great number of performed measurements. An increase in loads to 0.25*σ*_3max_—Figure 11 caused an evident increase in passing time of the ultrasonic wave for all models. The effect of previous original disturbances was found on nearly whole surfaces of the units. The greatest difference in results was observed near edges of masonry units. As in the primary phase, the coefficient of variation was minor and ranged from 1.0–1.3%. An increase in loads to 0.50*σ*_3max_ and 0.25*σ*_3max_—Figure 12 and Figure 13 produced a gradual increase in mean time of propagation, but did not cause apparent qualitative changes in maps presenting passing times. Similarly, coefficients of passing time of waves did not dramatically changes as the maximum value they reached was 1.4%.

## 5. Analysis of Test Results

On the basis of empirical relationships and those developed in the testing stage, an attempt was made to determine normal stresses in the tested models. The comprehensive approach based on all test results or the approach using a limited number of points was implemented for each model. In the first case, there were 315 (the model of series I) or 308 (the models of series II or III) measurement results for each step of loading. The calculations also included results for edges of the masonry units that demonstrated clear disturbances. The approach based on a limited number of points for determining stress involved only points located in the central area of the masonry units. That significantly limited the number of analyzed measuring points to 45 for model I, and 44 for models of series II and III. For successive levels of loading, the difference in passing time of the ultrasonic wave was calculated, and then acoustoelastic coefficient β_113_ was calculated from Equation (38). Finally, stress σ_3_ from the transformed relationship (36) was calculated. The obtained values of stress are presented in Table 5.

The obtained coefficients depended on apparent density of AAC of the order −0.0215–−0.0224 mm^2^/N. The values obtained for autoclaved aerated concrete aerated were many times greater than similarly determined acoustoelastic effect for metals [40] (*β*_113_ = −0.99 × 10^−5^–−2.06 × 10^−5^ mm^2^/N—steel, *β*_113_ = −7.75 × 10^−5^ mm^2^/N—aluminium, *β*_113_ = −1.88 × 10^−5^ mm^2^/N—copper). The determined stress values were similar only at relatively low stress values equal to 0.25*σ*_3max_ and 0.50*σ*_3max_. Maximum differences in stress determined using the EA method did not exceed 11% (model II-1). For stress values of the order of 0.75*σ*_3max_, the estimated values of non -destructive stress were considerably lower than those determined from destructive testing. Stress values were underrated by no more than 28%.

In the second approach based on the limited number of results for central areas of all masonry units, the procedure was similar to the first one. The location of measuring points in the central part of the masonry units was determined by analysing the maps of passing times illustrated in Figure 10, Figure 11, Figure 12 and Figure 13. Firstly, differentiation in passing time of ultrasonic waves was smaller in the central areas. Secondly, stress states in that area of masonry units was the most similar to stress states in the specimens 100 × 100 × 100 mm used to validate the AE method in stage I. In addition, the final aspect was purely practical because it was the easiest to determine centers of masonry units, apart from edge areas. For successive levels of loading, the difference in passing time of the ultrasonic wave was calculated. Then, acoustoelastic coefficient *β*_113_ was calculated from Equation (38), and finally stress values σ_3_ were calculated from the relationship (36). The obtained values of stress are presented in Table 6.

**Figure 14:**
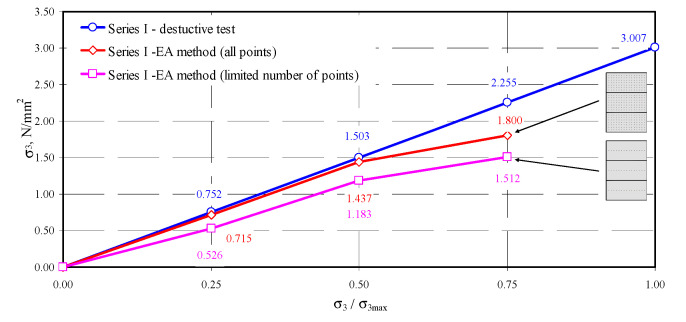
Measurement results of stress in the model I-1.

**Figure 15:**
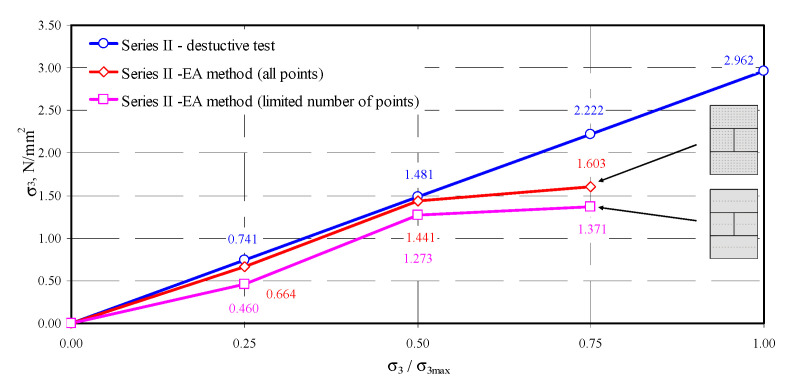
Measurement results of stress in the model II-1.

**Figure 16:**
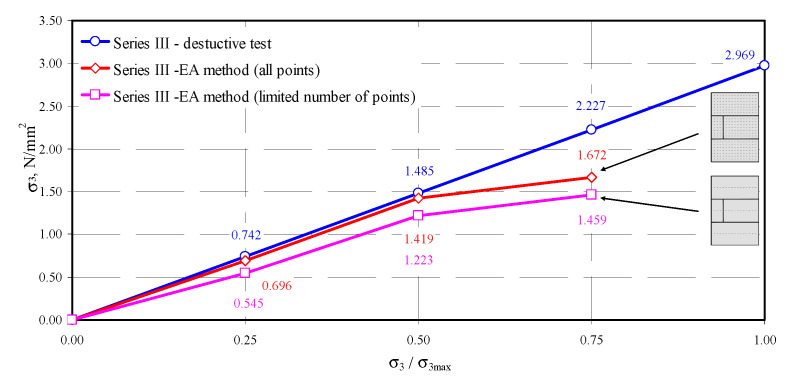
Measurement results of stress in the model III-1.

Using the approach of considerably decreased number of measuring points limited to central areas of the masonry units, much lower stress values were obtained. For the lowest level of stress of the order of 0.25 σ_3max_, stress calculated for the model II-1 with the AE method was lower by 60% than in destructive tests. Stress underestimation for other models I-1 and III-1 was at the level of 36–43%. At the stress level of 0.50*σ*_3max_, the underestimation of stress was at the lowest level of 16–21%. As in the case of a greater number of points, stress values determined by the EA method at the stress level of 0.75*σ*_3max_ were the least accurate. Calculated compressive stress differed by 49–62% from experimentally obtained values. Compared results from destructive testing and calculated results are shown in Figure 14, Figure 15 and Figure 16.

In conclusion, the most favorable results from measuring stress with the calibrated acoustoelastic method were obtained when all measuring points were used at stress levels within the range of 0–0.5*σ*_3max_. The determined stress values were lower than those from destructive testing small wall models. Considering the approach based on the limited number of points, underestimation of compressive stress was considerably greater. The greatest differences in both methods were found at the stress level of 0.75 *σ*_3max_, which resulted from an increase in effects of ultrasonic wave scattering, developing microcracks in AAC structure (invisible on the external surface of the models).

### Statistical Estimation of Stress in Walls

The practical application of that method requires further tests mainly on location of measuring points and their minimal number. However, assuming only measuring points for central area of each masonry unit are used to determine stress in the masonry, then boundary values of strength could be determined with the probability that the obtained results were not lower than experimentally obtained results. Only values from the range of 0–0.5*σ*_3max_ were used for the calculations. The selected range seems to be the most reasonable because at the operational stage force values in real walls can correspond to maximum stress of the order of 50% of the calculated compressive strength of the wall f_d_. Thus, load-carrying capacity of the real wall [43] depends not on absolute values of compressive force generating stress *σ*_3_, but on the stability expressed by the reduction factor for load-carrying capacity (*Φ*_1,2_ and *Φ*_2m_). Boundary values in confidence intervals of the mean value [53] (at *n* > 30 and unknown variance σ) were determined form the general relationship at the statistical significance α = 0.1:

(42) $$P\left(t_{p}-u_{1-\alpha /2}\frac{S}{\sqrt{n}}<t_{pcal}<t_{p}+u_{1-\alpha /2}\frac{S}{\sqrt{n}}\right)=1-\alpha$$

where: *t*_p_—mean time of wave propagation, *S*—standard deviation of propagation velocity for the specimen. $u_{1-\alpha /2}$—statistics with the random variable at the normal distribution N(0.1). When *n* < 30, the statistics $t_{1-\alpha /2}$ with the Student’s *t*-distribution and *n*-1 degrees of freedom should be applied.

Only the upper value of confidence interval is suitable for practical applications, which in this case can be associated with the quantile of the order of 95%. In other words, the upper limit of the confidence interval for the mean value was assumed because it is commonly used in the construction sector. Stress values were determined with the AE method using calculated values of passing time of the wave. The obtained results were compared with true mean stress values of the masonry wall. Values for upper confidence intervals for passing time *t*_pcal_ and calculated stress values *σ*_3cal_ using the AE method are presented in Table 7 and compared with stress results obtained from testing the models *σ*_3obs_. In that way, we obtain some estimation of the deviation between test and calculated results at the specified confidence level.

Taking into account the statistical estimation of stress, it was underestimated but values were significantly reduced. It can suggest with the probability of not greater than 5% that determination of stress in the walls from central areas of the masonry units with the slightest disturbances will cause underestimation of the mean stress at 0.25*σ*_3max_ by 18%, and at 0.50*σ*_3max_ by ca. 12%. That underestimation can be acceptable for masonry structures.

## 6. Conclusions

This paper describes theoretical bases of the acoustoelastic method (AE) which is one of the methods of detecting stress in structures using NDT techniques. That method consists of the correlation between stress in the material and velocity of the wave propagation. It is commonly used in ultrasonic tensometry to determine own stresses usually in machine parts. Using that method for other materials has not been widely discussed in the literature so far. No results from tests and analyses in concrete, not mentioning masonry, are available. This lack of interest in using this method can only be explained by measuring difficulties (significant dispersion of measurement results) caused by inhomogeneity of that material. This work presents an attempt to use the AE method for autoclaved aerated concrete. It is a porous material with high homogeneity and repeatability of parameters due to the production of this material on an industrial scale. This work supplements comprehensive material tests for autoclaved aerated concrete [11]. The tests were divided into two stages: Stage I involved the suggestion of the procedure and the determination of acoustoelastic coefficient *β*_113_ linking the propagation of the longitudinal ultrasonic wave c_p_ with normal stress *σ*_3_ acting towards the wave propagation. The standard cuboid specimens with the dimensions of 100 × 100 × 100 mm were used for calibration. The effect of density ρ and relative humidity w was included on the basis of testing AAC of different density using correlations presented in [11]. Those considerations resulted in formulating the relationship *β*_113_ (*ρ*). The proposed procedure was verified in stage II, where destructive tests were conducted on small masonry walls made of autoclaved aerated concrete (AAC) with a nominal density of 600 kg/m^3^. The models were divided into three series differing in the location of head joints in the masonry. Velocity of the ultrasonic wave propagation was measured for one model of each series at different values of compressive stress. The following stress levels were analyzed: 0.25*σ*_3max_, 0.50*σ*_3max_ and 0.70*σ*_3max_ because the range of the applied method was only limited to the elastic range. The performed measurements were used to determine values of acoustoelastic coefficients *β*_113_ = −0.0215–−0.0224, which were far lower than similarly determined acoustoelastic coefficients for metals. Mean stress values calculated with the proposed method using all measuring point for a given level (*n* = 308–315) were within the range of 93–96% of empirical values 0.25*σ*_3max_, 0.50*σ*_3max_. The highest underestimation of stress was found for the stress level of 0.75*σ*_3max_, for which the underestimation of mean stress values was equal to 24%. However, such a great number of measurements seem to be impractical for the applicable uses. Therefore, further analyses suggest determining stress values only on the basis of measurement results for central areas of each masonry unit. Then, the number of measuring points was significantly reduced to *n* = 45 and 44. As for all measuring points, the comparison indicated greater underestimation of the mean value of the order of 22–55%. It is not advantageous taking into account safety of the structure. Hence, it was decided to estimate the confidence interval of the mean value associated with the quantile of the order of 95%. Such a procedure caused the stress values were underestimated at the level of 12–18% within the stress range of 0–0.50*σ*_3max_. In summary:

(a) the acoustoelastic method (AE) can be used to determine stress in autoclaved aerated concrete,
(b) correlations were obtained that bind the value of acoustoelastic coefficient *β*_113_ as a function of density and moisture content in AAC,
(c) the effect of scattering of the ultrasonic wave in medium can be neglected when the coefficient *β*_113_ is applied,
(d) rather precise values of mean stress in the wall were determined on the basis of measured velocity of ultrasonic wave propagation at a high number of measuring points,
(e) reduced number of measuring points resulted in a significant underestimation of mean stress,
(f) determination of the quantile equal to 95% for passing time of the ultrasonic wave was used to estimate stress in the wall with the underestimation of the order of 12–18%, which can be considered as satisfactory.

The formulation of explicit recommendations to diagnose in-situ structures requires additional tests on slender walls to evaluate the impact of stability and works on improving the selection of measuring points. The proposed procedure for selecting measuring points limited to central parts of masonry units can be inaccurate for slender walls. Tests are going to be performed on the acoustoelastic coefficient in the wall with a one-side access using transverse waves to determine the acoustoelastic coefficient *β*_133_.

## Figures and Tables

**Table 1:** Fundamental characteristics of masonry units.

No.	Nominal Class of Density kg/m^3^	Density Range of AAC, kg/m^3^	No. of Specimens (Cores φ59 × 120 mm)	Mean Density *ρ*_0_, kg/m^3^ (C.O.V) acc. to [12]	Mean Modulus of Elasticity, E, N/mm^2^ (C.O.V)	Mean Poisson’s Ratio *ν*, (C.O.V)
1	400	375–446	6	397 (6%)	1516 (9.6%)	0.19 (7.9%)
2	500	462–532	6	492 (3%)	2039 (8.9%)	0.21 (8.7%)
3	600	562–619	6	599 (2%)	2886 (10.5%)	0.20 (8.5%)
4	700	655–725	6	674 (3%)	4778 (10.1%)	0.19 (9.2%)

**Table 2:** Test results for ultrasound velocity in AAC at various compressive stresses.

No.	Mean Density *ρ* (Nominal Class of Density) kg/m^3^	Mean Compressive Stress *σ*_3_, N/mm^2^	Mean Relative Compressive Stress *σ*_3_/*σ*_3max_	Mean Path Length *L*, mm	Mean Passing Time of Wave *T*, µs	Mean Ultrasound Velocity *c*_p_ = *L/t*, m/s	Standard Deviation, *s*, m/s	COV, %
1	2	3	4	5	6	7	8	9
1	397 (400)	0	0	100.2	53.5	^obs^*c*_p0_ = 1875	1.02	1.9%
2		0.75	0.27		55.7	1801	1.78	3.2%
3		1.33	0.48		57.3	1750	0.56	1.0%
4		2.08	0.75		60.4	1660	0.13	0.2%
5		2.58	0.93		60.9	1647	1.19	2.0%
6	492 (500)	0	0	100.3	53.0	^obs^*c*_p0_ = 1893	0.62	1.2%
7		0.83	0.24		54.3	1849	1.04	1.9%
8		1.66	0.48		57.1	1756	0.54	1.0%
9		2.59	0.75		58.2	1724	1.55	2.7%
10		3.33	0.96		59.3	1691	1.23	2.1%
11	599 (600)	0	0	100.4	49.5	^obs^*c*_p0_ = 2031	1.32	2.7%
12		1.25	0.24		51.7	1942	1.79	3.5%
13		2.58	0.50		52.9	1898	1.32	2.5%
14		3.92	0.75		54.6	1841	1.75	3.2%
15		5.00	0.96		53.9	1866	2.40	4.5%
16	674 (700)	0	0	100.2	45.1	^obs^*c*_p0_ = 2225	1.56	3.5%
17		2.00	0.24		46.5	2159	2.34	5.0%
18		4.17	0.50		47.5	2114	2.08	4.4%
19		6.17	0.74		48.3	2075	1.72	3.6%
20		8.17	0.98		48.6	2064	1.71	3.5%

**Table 3:** Test results for all models.

No.	Series	Model	Mean Density *ρ*_0_, kg/m^3^	Moisture Content *w*, %	Maximum Moisture Content (39) *w*, %	Compressive Stress Inducing Cracks *σ*_3cr_, N/mm^2^		Maximum Compressive Stress *σ*_3max_, N/mm^2^	
						of Model	Mean (COV)	of Model	Mean (COV)
1	2	3	4	5	6	7	8	9	10
1	I	I-1 *	594	6.0%	60.9%	2.93	2.89 (1.1%)	2.97	3.01 (1.3%)
2		I-2	589	4.5%	61.6%	2.87		3.04	
3		I-3	592	5.1%	61.2%	2.88		3.01	
4	II	II-1 *	588	6.0%	61.7%	3.00	2.95 (2.8%)	3.00	2.96 (2.6%)
5		II-2	597	4.9%	60.6%	2.85		2.87	
6		II-3	593	6.0%	61.1%	2.99		3.01	
7	III	III-1 *	594	5.1%	60.9%	2.97	2.90 (3.3%)	2.99	2.97 (1.9%)
8		III-2	587	5.5%	61.8%	2.79		2.90	
9		III-3	590	5.4%	61.4%	2.95		3.01	

*—models, for which the propagation of ultrasonic waves c_p_ was measured.

**Table 4:** Results from measuring propagation of ultrasonic waves.

Model	Number of Measuring Points in Each Step of Loading *n*	Passing Time of Ultrasonic Wave Under Various Levels of Loading *t*_p_, μs (COV)											
		0			0.25*σ*_3max_			0.50*σ*_3max_			0.75*σ*_3max_		
		*t*_pmin_	*t*_pmax_	*t*_pmv_	*t*_pmin_	*t*_pmax_	*t*_pmv_	*t*_pmin_	*t*_pmax_	*t*_pmv_	*t*_pmin_	*t*_pmax_	*t*_pmv_
1	2	3	4	5	6	7	8	9	10	11	12	13	14
I-1	315	86	94.2	90.8 (1.4%)	86.7	98.8	92.2 (1.3%)	90.5	99.2	93.9 (1.4%)	87.7	99.9	94.4 (1.4%)
II-1	308	82.2	92.9	89.2 (1.6%)	86.3	94.4	90.6 (1.2%)	89.0	97.4	92.2 (1.1%)	90.2	96.4	92.5 (1.1%)
III-1	308	85	92.9	88.8 (1.4%)	87.1	93.5	90.2 (1.2%)	88.8	95.1	91.6 (0.9%)	90.1	95.4	92.1 (0.9%)

**Table 5:** Results from calculating normal stress in the wall using all measuring points.

Model	Number of Measuring Points *n*	0.25*σ*_3max_			0.50*σ*_3max_			0.75*σ*_3max_		
		$\frac{\left(t_{p}-t_{p0}\right)}{t_{p0}}$	*β*_113_ mm^2^/N (38)	$\sigma_{3}=\frac{\left(t_{p}-t_{p0}\right)}{\beta_{113}\cdot t_{p0}}$ N/mm^2^ (36)	$\frac{\left(t_{p}-t_{p0}\right)}{t_{p0}}$	*β*_113_ mm^2^/N (38)	$\sigma_{3}=\frac{\left(t_{p}-t_{p0}\right)}{\beta_{113}\cdot t_{p0}}$ N/mm^2^ (36)	$\frac{\left(t_{p}-t_{p0}\right)}{t_{p0}}$	*β*_113_ mm^2^/N (38)	$\sigma_{3}=\frac{\left(t_{p}-t_{p0}\right)}{\beta_{113}\cdot t_{p0}}$ N/mm^2^ (36)
1	2	3	4	5	6	7	8	9	10	11
I-1	315	−0.0154	−0.0215	0.715	−0.0310	−0.0224	1.437	−0.0388	−0.0215	1.800
II-1	308	−0.0149	−0.0215	0.664	−0.0322	−0.0224	1.441	−0.0359	−0.0215	1.603
III-1	308	−0.0150	−0.0215	0.696	−0.0306	−0.0224	1.419	−0.0360	−0.0215	1.672

**Table 6:** Results from calculating normal stress in the wall using a limited number of measuring points.

Model	Number of Measuring Points *n*	0.25*σ*_3max_			0.50*σ*_3max_			0.75*σ*_3max_		
		$\frac{\left(t_{p}-t_{p0}\right)}{t_{p0}}$	*β*_113_ mm^2^/N (38)	$\sigma_{3}=\frac{\left(t_{p}-t_{p0}\right)}{\beta_{113}\cdot t_{p0}}$ N/mm^2^ (36)	$\frac{\left(t_{p}-t_{p0}\right)}{t_{p0}}$	*β*_113_ mm^2^/N (38)	$\sigma_{3}=\frac{\left(t_{p}-t_{p0}\right)}{\beta_{113}\cdot t_{p0}}$ N/mm^2^ (36)	$\frac{\left(t_{p}-t_{p0}\right)}{t_{p0}}$	*β*_113_ mm^2^/N (38)	$\sigma_{3}=\frac{\left(t_{p}-t_{p0}\right)}{\beta_{113}\cdot t_{p0}}$ N/mm^2^ (36)
1	2	3	4	5	6	7	8	9	10	11
I-1	45	−0.0113	−0.0215	0.526	−0.0255	−0.0215	1.183	−0.0326	−0.0215	1.512
II-1	44	−0.0103	−0.0224	0.460	−0.0285	−0.0224	1.273	−0.0307	−0.0224	1.371
III-1	44	−0.0117	−0.0215	0.545	−0.0263	−0.0215	1.223	−0.0314	−0.0215	1.459

**Table 7:** Compared results from tests and upper values of confidence intervals.

Model	Number of Measuring Points *n*	*u*_1−α/2_	0.25*σ*_3max_				0.50*σ*_3max_			
			$t_{\mathrm{pcal}}$	$\sigma_{3\mathrm{cal}}=\frac{\left(t_{pcal}-t_{p0}\right)}{\beta_{113}\cdot t_{p0}}$ N/mm^2^	σ_3obs_ N/mm^2^	$\frac{\sigma_{3obs}}{\sigma_{3cal}}$	$t_{\mathrm{pcal}}$	$\sigma_{3\mathrm{cal}}=\frac{\left(t_{pcal}-t_{p0}\right)}{\beta_{113}\cdot t_{p0}}$ N/mm^2^	σ_3obs_ N/mm^2^	$\frac{\sigma_{3obs}}{\sigma_{3cal}}$
1	2	3	4	5	6	7	8	9	10	11
I-1	45	1.645	92.6	0.656	0.752	1.15	93.9	1.329	1.503	1.13
II-1	44		90.8	0.595	0.741	1.24	92.4	1.37	1.481	1.08
III-1	44		90.3	0.640	0.742	1.16	91.7	1.302	1.485	1.14
on average:						1.18	on average:			1.12
